# An Enzymatic Assay for High-Throughput Screening of Cytidine-Producing Microbial Strains

**DOI:** 10.1371/journal.pone.0121612

**Published:** 2015-03-27

**Authors:** Huina Dong, Yongfei Liu, Xin Zu, Ning Li, Feiran Li, Dawei Zhang

**Affiliations:** 1 Tianjin Institute of Industrial Biotechnology, Chinese Academy of Sciences, Tianjin, 300308, China; 2 Key Laboratory of Systems Microbial Biotechnology, Chinese Academy of Sciences, Tianjin, 300308, China; 3 National Engineering Laboratory for Industrial Enzymes, Tianjin, 300308, China; 4 The Light Industry Technology and Engineering, School of Biological Engineering, Dalian Polytechnic University, Dalian, 116034, China; IPK, GERMANY

## Abstract

Cytidine is an industrially useful precursor for the production of antiviral compounds and a variety of industrial compounds. Interest in the microbial production of cytidine has grown recently and high-throughput screening of cytidine over-producers is an important approach in large-scale industrial production using microorganisms. An enzymatic assay for cytidine was developed combining cytidine deaminase (CDA) and indophenol method. CDA catalyzes the cleavage of cytidine to uridine and NH_3_, the latter of which can be accurately determined using the indophenol method. The assay was performed in 96-well plates and had a linear detection range of cytidine of 0.058 - 10 mM. This assay was used to determine the amount of cytidine in fermentation flasks and the results were compared with that of High Perfomance Liquid Chromatography (HPLC) method. The detection range of the CDA method is not as wide as that of the HPLC, furthermore the correlation factor of CDA method is not as high as that of HPLC. However, it was suitable for the detection of large numbers of crude samples and was applied to high-throughput screening for high cytidine-producing strains using 96-well deep-hole culture plates. This assay was proved to be simple, accurate, specific and suitable for cytidine detection and high-throughput screening of cytidine-producing strains in large numbers of samples (96 well or more).

## Introduction

Cytidine is an industrially important precursor for the production of some medicines, such as cytarabine hydrochloride and its intermediate ancitabine hydrochloride [1], 2'fluoro-2'-deoxy-cytidine and 2'fluoro-2'-deoxy-arabinocytidine [2]. Microbial production of cytidine has recently drawn more attention because of its efficiency and environmentally-friendly green production process compared to chemical production processes.

The enzymatic steps in de novo biosynthetic pathways of pyrimidine nucleotides such as UTP, CTP and dCTP are regulated in-vivo by feedback inhibition of key enzymes and by repression or attenuation of enzyme synthesis, by accumulation of end products or other metabolites [3, 4]. To accumulate a large amount of cytidine, cells must therefore be resistant to the feedback regulation, which means cells have to be metabolically modified. Several microorganisms (e.g. *Escherichia coli*, *Bacillus subtilis*) [5–7] have been modified for cytidine production by standard mutagenesis methods using ultraviolet radiation, diethyl sulfate treatment or low-energy ion mutations. After mutagenesis, the positive mutant strains are selected by toxic cytidine analogs.

The concentration of cytidine is usually measured using HPLC [7–9]. HPLC can accurately quantify trace amounts of cytidine, but requires pre-treatment to remove insoluble debris before analysis. This means that expensive and bulky instruments are required, and the numbers of samples that can be analyzed at once are limited. The lack of a fast and facile cytidine quantification assay made the selection process time-consuming and labor-intensive. Therefore, there is an urgent demand for the development of rapid and accurate screening methods after cell mutagenesis.

Enzymatic assays are attractive solutions [10] as multiple samples can be analyzed simultaneously without any specialized, bulky and expensive instruments. Cytidine deaminase (CDA, EC 3.5.4.5) converts cytidine to uridine and NH_3_ using a zinc-activated water or hydroxide in an initial hydrolytic attack on C4 of the substrate [11] (Equation (1)).

$${\text{Cytidine+H}}_{\text{2}}\text{0}\overset{\text{CDA}}{\underset{\text{pH7.4}}{\to }}\text{Uridine}+{\text{NH}}_{\text{3}}$$ (1)

It is a promising enzyme for the development of a cytidine-selective assay. Richards DA *et al* determined CDA activity through analysis of the product uridine using HPLC [12]. However, the measurement of ammonia formed the basis of conventional methods for the assay of cytidine deaminase. Several methods have been developed for ammonia analysis, such as ion-exchange method [13, 14], dry-film method using diffuse separation [15, 16], indophenol method (Berthelot method) [2, 17, 18] and microfluorescence assay using phthalaldehyde and mercaptoethanol [19, 20]. Enzymatic methods using glutamate dehydrogenase (GLDH) [21–23], L-glutamine synthetase (GS) [24] and an enzymatic cycling system composed of three enzymes: NAD synthetase (NADS), glucose dehydrogenase (GlcDH), and diaphorase (DI) [25] have also been used. In particular, the indophenol method has been widely used for clinical and food analyses [26].

Here we describe an assay detecting cytidine and increasing the efficiency of screening of high cytidine-producing strains based on CDA. This method combines CDA and the indophenol method, in which the variation of blue color can be monitored via the OD_630_. The high-throughput screening can be achieved in 96-well plates. We also verified this assay method by HPLC. This method was successfully applied to measuring the amount of cytidine in the broth of different cytidine-producing *Bacillus subtilis* strains cultured in 96-well deep-hole culture plate. The high-throughput screening of cytidine-producing strains is also discussed.

## Materials and Methods

### Materials and equipment

All chemical reagents were of analytical grade and purchased from Sigma-Aldrich (St Louis, MO, USA). Primerstar *Taq* polymerase was purchased from Takara, restriction endonuclease, T4 DNA ligase and their corresponding buffers were purchased from New England Biolabs (USA). 96-well microtiterplates were purchased from Nunc (Denmark). Ni-NTA agarose resins were supplied by GE Healthcare (USA) for His-tagged protein purification.

All polymerase chain reactions (PCR) were performed using a thermal cycler (DNA Engine; Bio-Rad, Hercules, CA, USA). Colorimetric assays were measured using a microtiterplate reader (SpectraMax M2e, Molecular Devices, Sunnyvale, CA, USA). HPLC analysis was performed using an Agilent 1260 (Agilent Technologies, Waldbronn, Germany).

### Plasmids, bacterial strains and media

Plasmid pET28a carrying an N-terminal His•Tag and an optional C-terminal His•Tag was purchased from Invitrogen (USA). The host bacterial *E*. *coli* DH5α and BL21 (DE3) were purchased from Trans-Gen Biotech Company for the construction, propagation and expression of plasmids. The cytidine-producing *B*. *subtilis* was reserved in our laboratory to amplify the *cdd* gene (encoded cytidine deaminase).

The Lysogeny Broth (LB) medium used contains (per liter) 10 g tryptone, 5 g yeast extract and 10 g NaCl. LB agar plates were prepared by adding 1.5% agar. M9 minimal salts medium contains (per liter) 12.8 g Na_2_HPO_4_·7H_2_O, 3 g KH_2_PO_4_, 0.5 g NaCl, 1 g (NH_2_)_2_CO, 1 mM MgSO_4_·7H_2_O, 0.1 mM CaCl_2_, 0.05 g tryptophan, micronutrient components (1 μM FeSO_4_·7H_2_O, 0.01 μM ZnSO_4_·7H_2_O, 0.08 μM MnCl_2_·4H_2_O, 0.4 μM H_3_BO_4_, 0.03 μM CoCl_2_·6H_2_O, 0.01 μM CuCl_2_·2H_2_O, and 3 nM Na_2_MoO_4_), appropriate amounts of glucose and 20 mg neomycin.

### Plasmid construction

Two primers were employed to amplify *cdd* from the genomic DNA of *B*. *subtilis* and designed as follows: Bs-cdd-F (5’- CGC **GGATCC** (*Bam*HI) ATGAACAGACAAGAATTAATAACAGAA-3’) and Bs-cdd-R (5’- CCG **GAATTC** (*Eco*RI) TTAAAGCTTTCGTTCGTCATGTAAATC-3’). The PCR product was purified from agarose gel, digested with *Bam*HI and *Eco*RI and subsequently inserted into the *Bam*HI*-Eco*RI site of pET28a vector to yield the pET28a-*cdd*. DH5α cells with plasmids were cultured aerobically at 37°C in LB medium or on LB agar plates with 50 mg/L Kanamycin. The constructed pET28a-*cdd* was expressed in *E*. *coli* BL21 (DE3).

### Expression and purification of CDA

Expression of CDA, *E*. *coli* BL21(DE3) including pET28a-*cdd* plasmid was induced for 2 h at OD_600_ of 0.6–0.8 in LB (contained 50 mg/L Kanamycin) by adding 1 mM Isopropyl β-D-1-thiogalactopyranoside (IPTG) [27]. Cells expressing CDA were harvested, cell pellets were suspended in lysis buffer (50 mM NaH_2_PO_4_, pH 8.0, 300 mM NaCl, 10 mM imidazole, 10 mM β-mercaptoethanol) and disrupted by sonication. The supernatant fractions were subjected to Ni-NTA agarose and equilibrated for 2 h at 4°C. By applying the mixture to the column for protein purification, the unbound proteins were washed off the column by washing buffer (50 mM NaH_2_PO_4_, pH 8.0, 300 mM NaCl, 25 mM imidazole, 10 mM β-mercaptoethanol). Bound proteins were eluted by elution buffer with high concentration of imidazole (50 mM NaH_2_PO_4_, pH 8.0, 300 mM NaCl, 250 mM imidazole, 10 mM β-mercaptoethanol). Purified proteins were dialyzed against a storage buffer (50 mM NaH_2_PO_4_, pH 8.0 and 1 mM DTT) overnight at 4°C. The purified enzyme was subsequently mixed with 40% glycerol and stored at -20°C before use.

### CDA assay for cytidine detection

The featured product of the cytidine assay used in this study is indophenol, which has a maximum absorbance at 630 nm.

$${\text{Cytidine+H}}_{\text{2}}\text{0}\overset{\text{CDA}}{\underset{\text{pH7.4}}{\to }}\text{Uridine}+{\text{NH}}_{\text{3}}$$ (1)

[28]

$${\text{NH}}_{\text{3}}\text{+NaOCl+phenol}\overset{\text{NaOH}}{\underset{\text{NaFe}{\left(\text{CN}\right)}_{\text{5}}\text{NO}}{\to }}\text{indophenol}$$ (2)

[17]

Standard cytidine (1M) was prepared in Milli-Q deionized water as a stock solution, the assay was performed in a 96-well plates. The total volume of the reaction mixture was 225 μL. First, 25 μL of the cytidine samples (fermentation broth or cytidine standard) were mixed with 100 μL of 0.01 M phosphate-buffered saline (PBS) and 0.06–0.3 U of the purified enzyme. The mixture was incubated for 20 min at 37°C before adding 50 μL reagent I (containing 20 mL/L phenol and 1 g/L sodium nitroprusside), 50 μL reagent II (containing 12.5 g/L sodium hydroxide and 30 mL/L sodium hypochlorite). The resulting mixture was incubated for 30 min at 37°C. For accurate detection using a microtiterplate reader and values of OD_630_ nm in the range of 0.2–0.9, the reaction mixture was diluted with water in proper ratio.

To improve the sensitivity of the assay, the concentrations of phenol, sodium nitroprusside, sodium hydroxide and sodium hypochlorite have been optimized. Absorbance values measured at the end of the reaction were used to construct cytidine standard curves. The different concentrations of cytidine used in different standard curves were obtained by diluting cytidine stock solution in ddH_2_O, LB medium, M9 medium and 10-fold diluted LB medium. The detection limit of the assay was defined as three times the standard deviation of cytidine-free blank samples (n = 20) [29]. All enzyme assays were performed in triplicates.

### Enzyme assay of CDA

Enzyme activity of CDA was expressed as the amount of ammonia produced per minute. One unit is equivalent to the release of 1 μmol ammonia per minute at 37°C (1U = 1μmol/min). The specific enzyme activity was calculated as follows: Specific activity = *V*_max_ ·V_r_ / m (V_r_: the volume of reaction; m: the amount of protein). The specific enzyme activity unit is U/mg. Protein determination was performed by 2-D quant kit (General Electric Company), using bovine serum albumin as standard.

The enzyme activity was measured as described in section 2.5. In the assay, 25 μL of 100 mM cytidine standard was mixed with 100 μL 0.01 M PBS (pH7.4) and 0.006 U of the purified CDA. The data used to calculate specific activity, *k*_*cat*_ and *k*_cat_/*K*_m_were listed in S1 Table.

### Substrate specificity for the CDA assay

The effects of different ribonucleosides and deoxyribonucleosides were examined in the CDA assay. The reaction contained 2 mM ribonucleosides or deoxyribonucleosides and the assay was performed as described above.

### Fermentation and analysis of cytidine production

We wanted to investigate whether this assay could be applied to fermentative processes as a rapid and accurate tool for cytidine detection. Mineral salts and nutrients being used in conventional microbiological culture media were examined to investigate their effects on the CDA assay. The reaction contained 2 mM cytidine and the assay was performed as described in section 2.5.

A batch fermentation was processed in M9 minimal salts medium, adding 4% glucose. The fermentation was performed in 250-mL Erlenmeyer flasks with 50 mL M9 medium. The flasks were kept in a shaker incubator at 220 rpm and 37°C for 48 h. The biomass concentration was determined by OD_600_ in a UV spectrophotometer. The concentration of cytidine in the cell-free culture supernatant was measured using the CDA assay and HPLC. Samples, which were harvested at a certain time of fermentation, were centrifuged at 13,000 rpm for 2 min at 20°C. The supernatants were filtered through a 0.22-μm membrane (⌀ = 0.22 μm) filter. To separate the compounds, aliquots (10 μL) were injected and analyzed using an Agilent 1260 HPLC with a 5C18-250A column (Agilent, 4.6mm id ×250 mm) thermostated at 35°C. The mobile phase consists of water: acetonitrile (95: 5 [v/v]) at a flow rate of 0.8 mL/min. Analytes were detected at 280 nm.

## Results

### Expression and purification of CDA in *E*. *coli*

**Fig 1:**
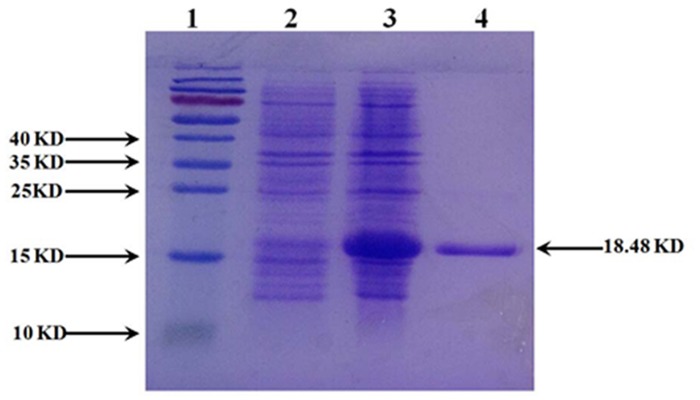
SDS-PAGE analysis of the CDA expression. *E*. *coli* BL21 (DE3) cells containing pET-28a-*cdd* were grown and induced with 1 mM IPTG. The cells were sonicated and then centrifuged to divide into two fractions, soluble and insoluble fractions. Soluble fraction was then purified using Ni-NTA agarose. Lane 1, size markers; Lane 2, total proteins of the uninduced cells; Lane 3, total proteins of the IPTG-induced cells; Lane 4, purified protein of CDA.

The coding region of *cdd* gene from *B*. *subtilis* was expressed in *E*. *coli* using pET28a expression system. After a 2-h induction of the BL21 (DE3) strain of *E*. *coli* harboring pET28a-*cdd* with 1 mM IPTG, an 18.48-kDa soluble protein was expressed. The level of protein expression was about 50% of the total cellular protein mass. The molecular mass of the expressed protein matched with the calculated value (14.96 kDa) based on the amino acid sequence deduced from the nt sequence. The CDA protein was purified as the His-tagged fusion protein (18.48 kDa), which resulted in a CDA preparation with an approximately 95% purity (Fig. 1) judged by SDS-PAGE. Purified CDA was used for the construction of enzymatic assays to detect cytidine as below.

### Activity determination of CDA

**Fig 2:**
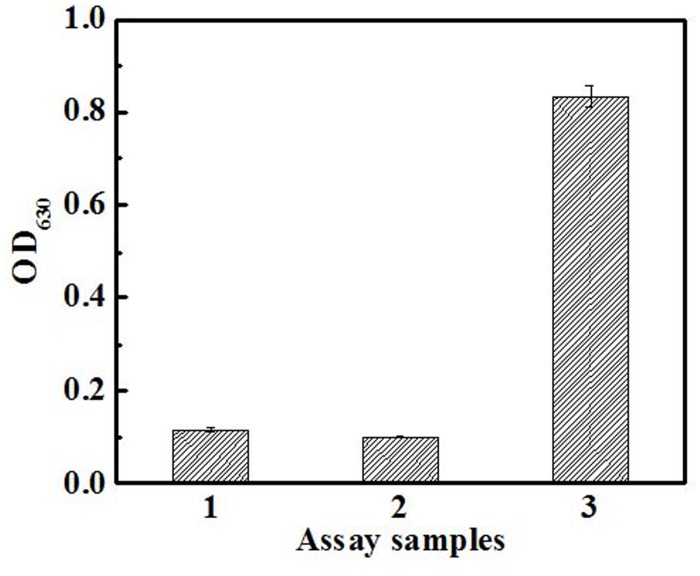
Activity determination of CDA. 1- reaction reagent without CDA and cytidine; 2- reaction reagent with CDA and without cytidine; 3- reaction reagent with CDA and cytidine. The concentration of cytidine is 1 mmol/L. The experiments were performed in triplicate.

Activity determination of CDA was conducted according to the method mentioned in section 2.5. The purified CDA had a strong deamination activity to cytidine in comparison with the control experiments, in the absence of CDA, as shown in Fig. 2. Although blank samples generated a slight background signal, the purified CDA still showed significant activity. The specific activity of the purified CDA was estimated to be 184.5 U/mg. The steady-state saturation kinetics of CDA was investigated using cytidine as the variable substrate. *K*_m_ of CDA was 1.009±0.067 mM, *k*_*cat*_ value was 56.8 s^-1^ and *k*_cat_/*K*_m_ was 5.63×10^4^ L/(M·s).

The specific activity is comparable in magnitude with that reported (238 U/mg) by Carlow *et al* [30] and that reported (231.8 U/mg) for *B*. *subtilis* ED 213 [31]. The *K*_m_ value was about 4.5-fold of that reported by Johansson *et al* [32].

### Determination of cytidine based on CDA

**Fig 3:**
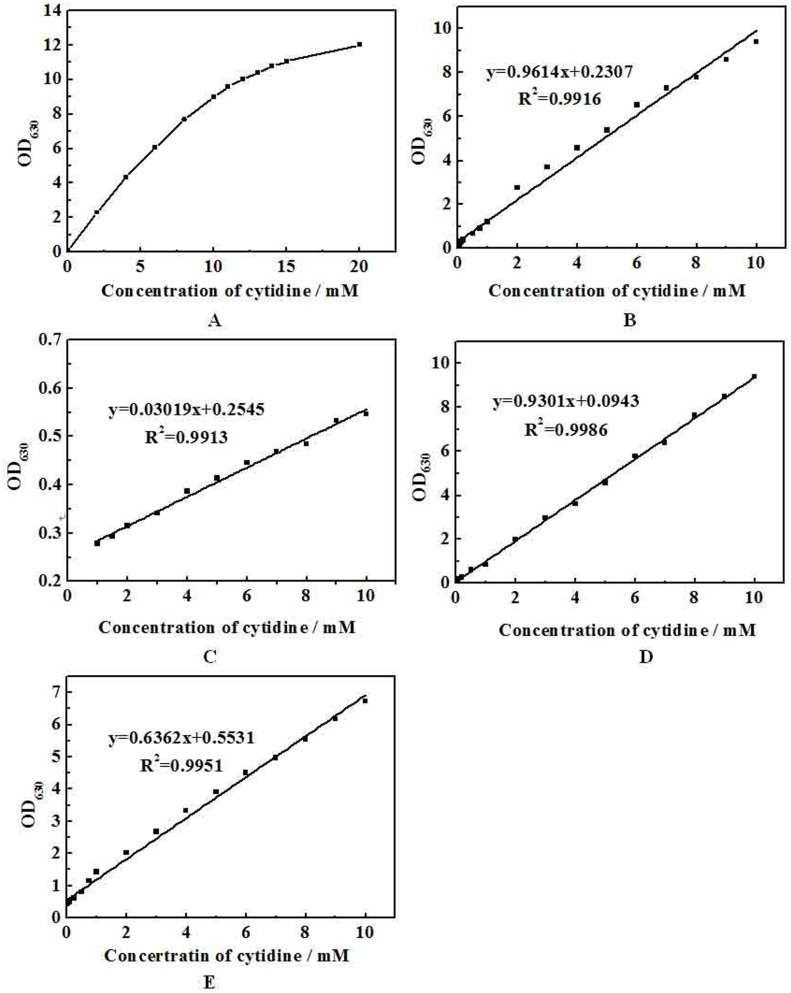
(A). Effect of different cytidine concentrations on CDA assay in H_2_O. Cytidine standard curves were conducted under standard reaction conditions in section 2.5 in H_2_O (B), LB medium (C), M9 medium (D) and 10-fold diluted LB medium (E), respectively. Each plot represents the average of three samples. Absorbance was measured using a microplate reader.

The cytidine assay was developed by coupling CDA with the indophenol method and conducted according to the method in section 2.5. The resulting indophenol had a maximum absorption at 630 nm. The addition of cytidine resulted in a proportional color development, which was translated to a linear standard curve (Fig. 3B-E). The upper range of the assay (in water) was also been explored (Fig. 3A). The increase of absorbance begins to fall when the concentration of cytidine is higher than 10 mM. The linear range and detection limit in H_2_O, LB medium, M9 medium and 10-fold diluted LB medium were listed in Table 1. The regular M9 medium contains (NH_4_)_2_SO_4_, which has significant influence on the reaction. Therefore, we replaced it with urea. The modified M9 medium enabled highly sensitive detection of analytes, even though containing a low concentration of cytidine. LB medium has negative influence on the assay, but the influence decreased as the medium was diluted (Fig. 3E). This result demonstrated that an accurate concentration of cytidine can be determined specifically using this colorimetric assay. Therefore, the fermentation samples in LB medium should be diluted before CDA reaction. The dilution step is necessary when using complex medium.

**Table 1:** Summary of parameters of the cytidine assay by CDA.

	H_2_O	LB medium	M9 medium	10 fold dilutedLB medium
Linear range of cytidine concentrations (mM)	0.05–10	2.5–10	0.058–10	0.102–10
R^2^	0.9931	0.9913	0.9965	0.9951
Detection limit (mM)	0.05	2.5	0.058	0.102

### Substrate specificity test of CDA

The deamination activity of CDA was examined with various ribonucleosides, deoxyribonucleosides and nucleotide-related substances. The relative absorbance are presented in Table 2. The cytidine deaminase catalyzes the deamination of deoxycytidine, 5-f1uorocytidine, 5-fluorodeoxycytidine, Cytosine-β-D-arabinofuranoside (Ara-C), 5-methylcytidine, azadeoxycytidine and 5-iodocytidine as well as cytidine. It showed no deamination activity with other kinds of ribonucleosides and deoxyribonucleosides, especially uridine and uracil, which are the main by-products in the fermentation of the cytidine-producing strain. Park *et al*. (31) reported the CDA from *B*. *subtilis* ED 213, which catalyze the deamination of deoxycytidine, 5-methylcytidine and 5-f1uorodeoxycytidine as well as cytidine, but the CDA showed no deamination activity with Ara-C, 5-iodocytidine and azadeoxycytidine.

**Table 2:** Substrate specificity of the CDA.

Substrate (2 mM)	Relative activity (%)	Substrate (2 mM)	Relative activity (%)
Cytidine	100	Uridine	0
Deoxycytidine	110	Adenosine	0
5-Fluorocytidine	81.6	Guanosine	0
5-fluorodeoxycytidine	80.1	Inosine	0
Cytosine-β-D-arabinofuranoside	79.5	Thymidine	0
5-methylcytidine	68.5	Deoxyuridine	0
Azadeoxycytidine	47	Deoxyadenosine	0
5-Iodocytidine	15.3	Deoxyguanosine	0
Uracil	0	Deoxyinosine	0
Cytosine	0	6-Azauracil	0

### Effect of medium components on the CDA assay

Various components in common media, nutrition components, precursors and by-products might cause fluctuations in the cytidine assay, so the effects of those components were studied (Table 3). Carbon resource in the medium, e.g. lactose, maltose, sucrose, glucose, xylose and arabinose, caused no inhibition of enzyme activity. In the CDA assay based on the indophenol method, many amino acids suppress development of blue color in deferent levels due to their primary or secondary amino groups [17]. Almost all multiple nutrition components, such as wort extract, tryptone, yeast extract and yeast powder, had influence on the accuracy of the CDA assay (77.3–126.9%). The error may be caused by endogenous components with functional amino groups. Some metal ions also had an effect on the cytidine assay at high concentrations, especially Zn^2+^. However, influence of metal ions at low concentrations can be ignored (data not shown).

**Table 3:** Effect of medium components on the cytidine assay.

Compound	Concentration (mM)	% absorbance	Compound	Concentration (g/L)	% absorbance	Compound	Concentration (mM)	% absorbance
Mineral salts			Nutritional components			Amino acids		
NaCl	10	99.1	Wort extract	25	126.9	Aspartic acid	2	2
ZnCl_2_	10	213.2	Tryptone	10	77.3	Cysteine	2	0
CoCl_2_	10	127.5	Yeast extract	5	85.5	Glutamate	2	61.6
FeCl_3_	10	92.4	Beef powder	5	112	Histidine	2	0
CuCl_2_	10	101.8	Lactose	10	86.8	Isoleucine	2	16.6
CaSO_4_	10	91.9	Maltose	10	110.3	Leucine	2	87.7
MnSO_4_	10	83.2	Sucrose	10	90.5	Lysine	2	23.4
Na_2_SO_4_	10	85.9	D-glucose	10	102.1	Methionine	2	0
FeSO_4_	10	109.7	D-xylose	10	98.9	Phenylalanine	2	2.23
CuSO_4_	10	87.9	L-arabinose	10	97.4	Tryptophan	2	5.8
Sodium acetate	10	115.6	D-Mannitol	10	89.7	Tyrosine	2	63.5
NaHCO_3_	10	99.1	D-Sorbitol	10	88.1	Valine	2	17.3
NaNO_2_	10	96.4	Urea	10	96.8			
Citric acid	10	81.8	Betaine	10	64.4			
Sodium citrate	10	88.6						
Calcium carbonate	10	89.9						

Note: Values reported in the table were the average of three parallel determinations. The absorbance was reported as a percentage of that obtained with cytidine, (2 mM) dissolved in water, i.e., ((absorbance with cytidine + test compound)/absorbance with cytidine alone) × 100%. A value of 100 means no interference; a value of 0 means total interference, i.e., no color formation at all, and values greater than 100 mean the test compound enhances the absorbance of the solution.

### Comparison of the CDA-based assay with HPLC method

**Fig 4:**
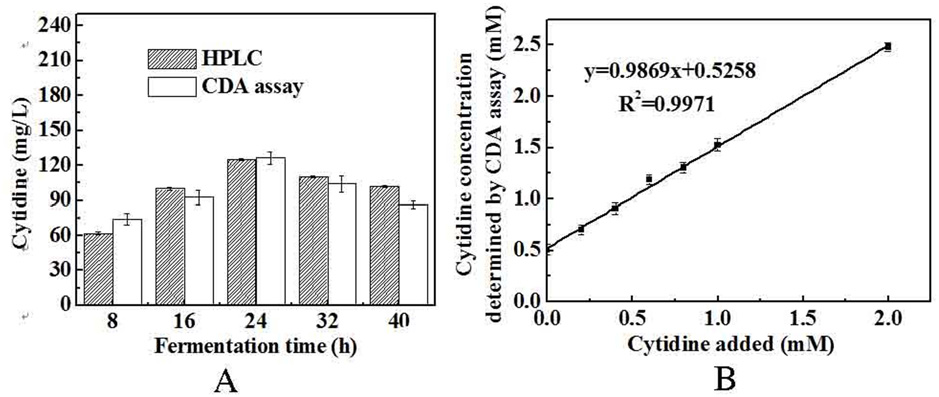
(A) Cytidine concentrations in fermentation flasks were determined using the CDA assay and HPLC. Samples were collected from the fermentation flasks every 8 h from 8 h to 40 h. (B). The correlation between cytidine added in fermentation broths and that detected by CDA assay.

The CDA assay mentioned in section 2.5 was evaluated by comparing determination results with that of HPLC. A batch fermentation with cytidine-producing strain was performed and samples were collected every 8h. Cytidine concentrations in fermentation samples were measured by both CDA assay and HPLC. Results showed that values of two methods fit well (Fig. 4A and S1 Fig.). Due to the complex nature of the fermentation medium composition and cell metabolism, we believed that some fermentation products may have some impact on the quality of the cytidine assay. Therefore, a blank control experiment should be performed to decrease the error. To detect cytidine for screening cytidine-production strains, samples were chosen at 24 h by considering fermentation time, by-products and other metabolites produced in fermentation.

The reliability of the CDA assay was further supported by adding 0–2 mM cytidine to the fermentation broth at 24 h. The assay estimated the intrinsic cytidine concentration in the fermentation sample to be 0.52±0.03 mM. This value was consistent with that estimated by HPLC, 0.50±0.004 mM, confirming the accuracy of the CDA assay in biological samples. As shown in Fig. 4B, high linearity (R^2^ = 0.9971) was obtained between the concentration of cytidine added in the fermentation broth and the cytidine concentration determined by CDA. From the slope of the linear correlation, the recovery of cytidine by CDA determination was 98.69%. It can be identified that this assay was accurate and reliable for determining cytidine concentration in fermentation flasks.

### Screening of cytidine-producing strains

To verify whether this CDA assay can be used to quantify the amount of cytidine produced by different bacterial strains at once, a cytidine-producing strain was treated with 402 nm laser for 3 minutes and isolated on LB agar plate. Then, 95 randomly picked colonies were cultured in a 96-well deep-hole culture plate with a control strain. Theses strains were further screened using ADA assay with 96-well plates.

**Fig 5:**
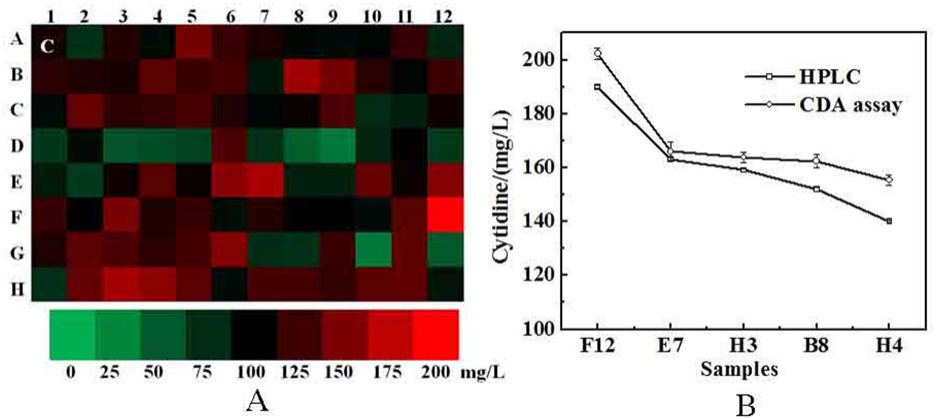
Screening high cytidine-producing strains using CDA assay. (A) Production of cytidine in a 96-well deep-hole culture plate from randomly picked mutation strains. The letters and numbers represented row and column number of the 96-well deep-hole culture plate respectively. C: Control, A1 represented for cytidine concentration of parent strain. (B) Top five samples (F12, E7, H3, B8 and H4) determined using CDA assay was chosen to detect cytidine by HPLC. CDA assay and HPLC had a positive correlation on cytidine concentration. Each experiment runs in triplicate.

The screening results are shown in Fig. 5A and Table 4. The broth of the top five cytidine-producing strains (F12, E7, H3, B8 and H4), in which the cytidine concentrations were determined using the CDA assay, were chosen to detect cytidine by HPLC along with the parent strain. As shown in Fig. 5B, both the CDA assay and the HPLC method showed a positive correlation on the amount of cytidine. Although the CDA assay was not so accurate for high-throughput screening in a 96-well deep-hole culture plate, the results illustrate that the CDA assay is still a good method to exclude more than 90% of the low-yielding strains and can be reliably applied to a high-throughput screening method for cytidine over-producing strains.

**Table 4:** Cytidine production of screening strains in 96-well deep-hole culture plate.

Cytidine concentration (mg/L)	1	2	3	4	5	6	7	8	9	10	11	12
A	109.2±5.9	73.6±4.2	114.1±3.2	94.8±3.8	146.4±4.9	122.6±4.7	110.2±4.5	96.6±3.9	94±4.9	97.2±5.3	121.3±4.9	78.6±4.1
B	116.9±4.5	112.2±5.3	109.6±3.7	136.7±7.5	123±3.3	125.9±5.0	87.9±3.6	162.5±5.4	144.8±4.7	115.4±4.8	97.3±3.7	124.1±5.2
C	96.1±5.4	139.3±5.8	117.7±4.3	120±7.3	127.8±4.2	111.5±4.6	97.1±6.1	104.1±3.9	129.3±5.5	76.4±3.9	83.9±4.2	105.9±4.9
D	69.3±7.8	95.2±6.1	53±5.1	56.8±6.0	62.7±3.6	130.1±3.7	73.2±3.4	47.1±3.6	31±4.1	79.8±5.0	102.2±3.5	66.5±3.0
E	86.3±6.2	67.6±6.8	105.7±4.3	134.1±4.4	103.6±3.5	153.8±5.3	166±7.2	83.3±3.8	82.3±.3.9	139.3±6.3	105±5.2	151.6±5.6
F	121±3.8	100.6±5.5	147.7±4.4	113.2±5.9	121.9±5.0	93.1±4.8	114.8±2.8	100.9±5.1	99.4±3.5	94.3±6.8	134.5±5.8	202.3±6.4
G	112.4±6.4	135.1±3.9	129.2±6.5	116.4±5.4	126.5±5.1	152.7±5.4	75.6±3.1	72.6±4.3	123.5±4.0	30.8±4.1	136.3±4.6	50.6±3.9
H	73.3±5.7	138.9±6.9	163.8±6.6	155.4±6.7	133.8±3.9	94.5±3.7	127.4±4.1	126.4±5.2	119.5±5.3	134.3±3.1	137.5±3.8	89.3±4.2

## Discussion

Cytidine is an important drug precursor and food ingredient. The current demand for cytidine provides a strong impetus to improve its production capacity and reduce its production cost. The traditional cytidine production is mainly through chemical synthesis, which uses cytosine and ribose or uridine as the main raw materials. However, this method involves many reaction steps, harsh reaction conditions, complicated operations, environmental pollution and high cost feedstock. Microbial production of cytidine is much more efficient when compared with chemical synthesis methods, such as low costs, high yield, short cycle time and easy fermentation control.

Up to now, the strain used in microbial producing of cytidine was mainly obtained through mutagenesis by physical or chemical methods. The disadvantages of being expensive, laborious and non-high-throughput motivated us to find a high-throughput screening method. As up to now there is no accurate, sensitive and convenient assay for cytidine detection.

In this study, we developed a simple and robust assay for cytidine by coupling CDA to the indophenol method. The enzymes used in the assay can be readily obtained by CDA over-expression in *E*. *coli*. The assay uses a simple detection method only requiring CDA in the assay mixture. It also has a low detection limit and is highly sensitive to low concentrations of substrate.

In general, the key enzymes in the de novo biosynthetic pathways of pyrimidine nucleotides are regulated by feedback inhibition and by repression and/or attenuation of enzyme synthesis by the accumulation of end products or other metabolites [4]. Therefore, pyrimidine nucleosides such as deoxycytidine can be synthesized in cells, however, are almost impossible to accumulate in the medium. Other cytidine-related substances such as 5-f1uorocytidine, 5-fluorodeoxycytidine, Ara-C, 5-methylcytidine, azadeoxycytidine and 5-iodocytidine can’t be synthesized by the cells. Although CDA catalyzes the deamination of these cytidine-related nucleotides, it can also be used to determine the amount of cytidine in fermentation.

Compared with the traditional qualitative and quantitative detection of cytidine using chromatographic methods such as HPLC, the reported assay is high-throughput, quick, sensitive and highly adaptable. The fluorescence assay[19] with sensitivity up to picomole quantities of ammonia in serum has low detection limit and linear relationship. However, the detection range (from 1 to 100 nmoles of ammonia) is too narrow for detecting cytidine in fermentation broth in large numbers of crude samples.

In minimum medium, the CDA assay based on the indophenol method can detect the amount of cytidine in the range of 0.058–10 mM. The sensitivity of this assay decreases significantly in complex media containing yeast extract, peptone, tryptone or beef powder. This is because they all contain compounds with primary or secondary amino groups that strongly suppress the development of blue color [17] in the indophenol method. Several amino acids and some compounds such as sodium sulfide, ascorbic acid and dimethyl sulfoxide mentioned by Ngo *et al* [17] also will depress the color in the indophenol method and CDA assay. However, few compounds mention above will be contained in fermentation broth. The diluted complex medium will have little effect on the CDA assay. The CDA assay allows easy detection of cytidine in the fermentation broth without any pre-treatment.

The result of this CDA assay for cytidine in the fermentation broth differs from the HPLC result, indicating that there are other metabolic substances affecting the assay; e.g. ammonia in the fermentation broth samples. Therefore, blank control experiments should be done to eliminate any background interference. As by-products from cytidine-producing strains interfere with the assay, an appropriate fermentation time should be chosen to screen cytidine-producing strains, such as 24 h. This can not only eliminate the influence, but also shorten the screening period.

The indophenol method has been widely used to quantify urea after splitting it enzymatically with urease [33] and other substrates [34, 35], and used in determining of deaminase activity [36]. This method can be easily adjusted to detect other metabolites or substances using a suitable deaminase. Although in this case the CDA was coupled to the indophenol method, the coupling partner could be many other enzymes (e.g. GLDH), or assay determining ammonia concentrations. These assays can also be used to quantify other biomolecules based on a suitable deaminase. Deoxycytidine and cytidine are equally good substrates for CDA, which means that the methods based on CDA are also suitable for high-throughput screening of deoxycytidine-producing strains.

## Supporting Information

S1 Fig HPLC trace of cytidine of fermentation broth at 8 hours. Cytidine: t = 3.903, uracil: t = 5.265, uridine: t = 6.776
(TIF)

S1 Table The parameters for calculating specific enzyme activity, *k*_cat_ and *k*_cat_/*K*_m_. Note: Vr: the volume of reaction; m: the amount of protein
(DOC)
