# Supplementary figures and images for: An Enzymatic Assay for High-Throughput Screening of Cytidine-Producing Microbial Strains
Source: PLoS One. 2015 Mar 27;10(3):e0121612. doi: 10.1371/journal.pone.0121612 (PMC4376533; doi:10.1371/journal.pone.0121612)

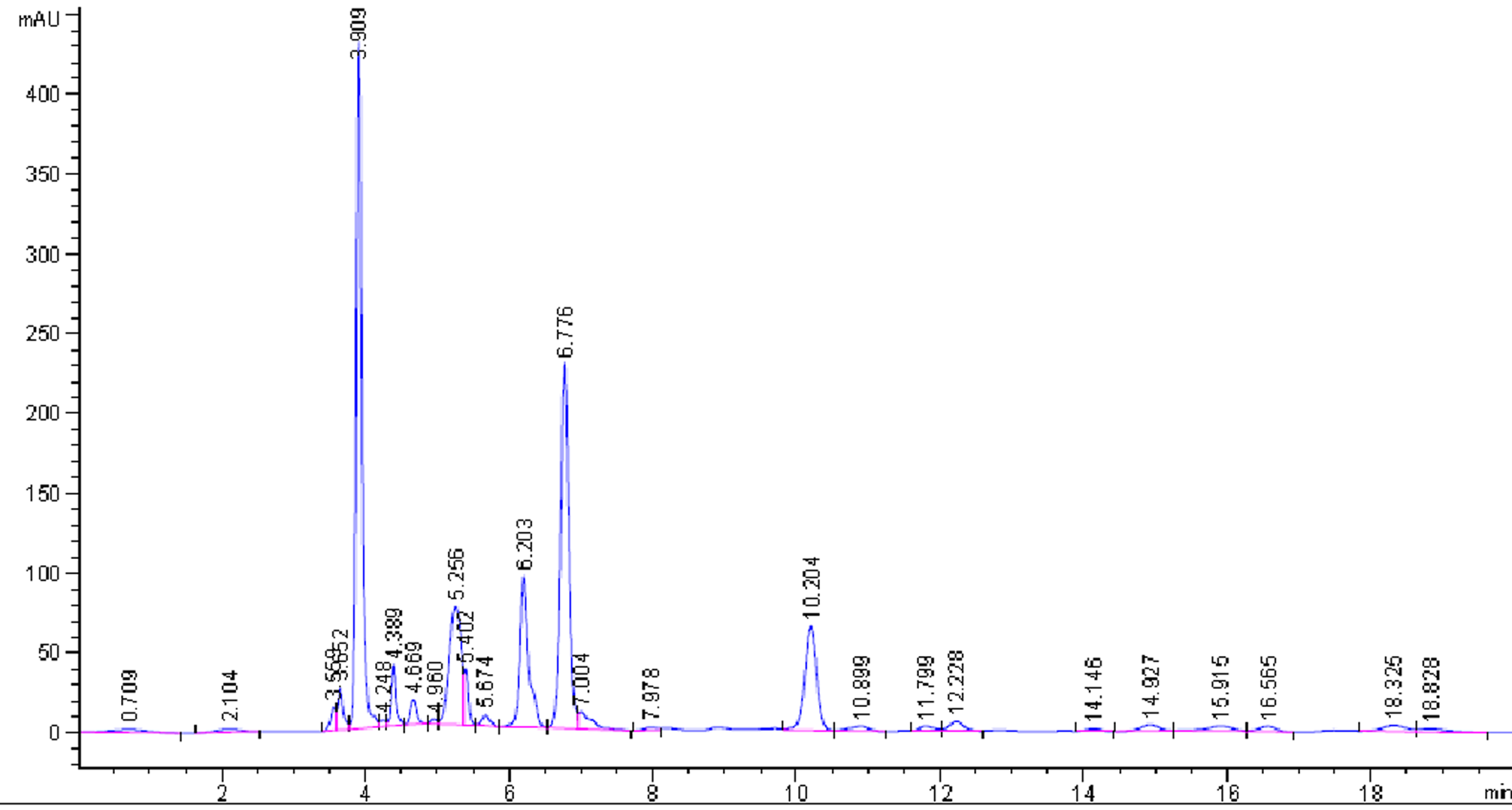

Supplement: S1 Fig — Cytidine: t = 3.903, uracil: t = 5.265, uridine: t = 6.776 (TIF) [file pone.0121612.s001.tif]
