# Supplementary material for: An Enzymatic Assay for High-Throughput Screening of Cytidine-Producing Microbial Strains
Source: PLoS One. 2015 Mar 27;10(3):e0121612. doi: 10.1371/journal.pone.0121612 (PMC4376533; doi:10.1371/journal.pone.0121612)
Supplement: S1 Table — Note: Vr: the volume of reaction; m: the amount of protein (DOC) [file pone.0121612.s002.doc]

**Supporting information**

**S1** **Table** The parameters for calculating specific enzyme activity, *k_cat_* and *k*_cat_/*K*_m_

| Parameters | Values |
| --- | --- |
| V_max_ | 0.0428±0.0052 mmol/(L·min) |
| V_r_ | 125 uL |
| m | 0.029 ug |
| MW | 18480 Da |
| K_m_ | 1.009±0.067 mmol/L |

Note: V_r_：the volume of reaction; m: the amount of protein
